# Diagnostic performance of hybrid cardiac SPECT/CT imaging for patients with takotsubo cardiomyopathy

**DOI:** 10.1186/s41824-017-0023-x

**Published:** 2018-03-05

**Authors:** Yasuro Sugihara, Yoshimitsu Fukushima, Shin-ichiro Kumita, Hitoshi Takano, Wataru Shimizu

**Affiliations:** 1 Department of Clinical Radiology, Nippon Medical School Graduate School of Medicine, 1-1-5 Sendagi, Bunkyo-ku, Tokyo, 113-0022 Japan; 2 Department of Cardiovascular Medicine, Nippon Medical School Graduate School of Medicine, 1-1-5 Sendagi, Bunkyo-ward, Tokyo, 113-0022 Japan

**Keywords:** Diagnostic performance, Takotsubo cardiomyopathy, Hybrid cardiac SPECT/CT

## Abstract

**Background:**

The diagnostic performance of SPECT-only imaging for takotsubo cardiomyopathy (TC) is limited due to the lack of coronary artery distribution information. The aim of this study was to evaluate the diagnostic performance of hybrid cardiac SPECT/CT for patients with TC or acute coronary syndrome (ACS).

**Methods:**

Twenty-two patients with apical ballooning left ventricular (LV) dysfunction who underwent cardiac perfusion SPECT using ^99m^Tc-methoxy-isobutyl-isonitrile (MIBI), cardiac fatty-acid metabolism SPECT using ^123^I–beta-methyl-P-iodophenyl-pentadecanoic acid (BMIPP), cardiac CT, and hybrid cardiac SPECT/CT imaging were analyzed. On the SPECT images, MIBI and BMIPP defects were quantified using a 17-segment model with a 5-point grading system and a summed MIBI defect score (SMDS), summed BMIPP defect score (SBDS), and summed mismatch score (SMS) were calculated. Furthermore, apical and non-apical MDS, BDS, and mismatch scores (A- and NA-MDS, A- and NA-BDS, and A- and NA-MS) were calculated. These quantitative scores were compared between the TC (*n* = 11) and ACS (n = 11) groups, and the diagnostic performances of SPECT-only imaging and hybrid SPECT/CT imaging were compared. For all patients, the diagnoses of both SPECT-only and SPECT/CT imaging were categorized as TC: SPECT accumulation defects around apical LV wall deviated from the actual coronary artery territories, equivocal: unclear relationship of accumulation defects and the coronary artery territories, or non-TC: accumulation defects coincided with the coronary artery territories.

**Results:**

SMDS and SBDS did not significantly differ between TC and ACS patients [SMDS: 5 (3–7) vs. 8 (4–16), *p* = 0.216; SBDS: 10 (8–12) vs. 18 (9–24), *p* = 0.354]. While A-MDS and A-BDS did not significantly differ between patients with TC and ACS (*p* = 0.567 and *p* = 0.386, respectively), NA-MDS and NA-BDS were significantly lower for patients with TC (*p* = 0.022 and *p* = 0.022, respectively). Compared with SPECT-only imaging (sensitivity: 30% and specificity: 81%), hybrid SPECT/CT imaging showed a higher accuracy (sensitivity: 90% and specificity: 100%) for the diagnosis of TC.

**Conclusions:**

Hybrid cardiac SPECT/CT imaging may have superior diagnostic performance compared with SPECT-only imaging for patients with TC.

## Background

Takotsubo cardiomyopathy (TC) is a common cardiac syndrome characterized by a transient left ventricular (LV) wall motion abnormality with apical hypokinesis and basal hyperkinesis, ST-segment elevation in electrocardiograms (ECG), slight myocardial enzymatic release, and no involvement of obstructive coronary artery disease (CAD) (Kawai et al. 2000, 2007; Kurisu and Kihara 2012). While the distinct pathophysiology of TC is still uncertain, it is likely multifactorial, including microvascular dysfunction, endocrine abnormality, and abnormal nervous responses to stressful events (Barletta et al. 2009). Furthermore, the diagnostic strategy for patients with TC is not adequately established (Ito et al. 2003; Kurisu et al. 2003; Matsuo et al. 2014).

Invasive coronary angiography (ICA), the most common diagnostic imaging modality for TC, is useful for ruling out obstructive CAD. However, this modality cannot reliably distinguish TC from acute coronary syndrome (ACS) caused by distal left anterior descending artery (LAD) vasospasms (Tsuchihashi et al. 2001; Prasad et al. 2008). Other modalities, cardiac perfusion single-photon emission computed tomography (SPECT) and cardiac fatty-acid metabolism SPECT with ^123^I–beta-methyl-P-iodophenyl-pentadecanoic acid (BMIPP), a branched-chain fatty-acid, can detect myocardial damage in TC (Hachamovitch et al. 2003; Tamaki et al. 1992; Kawai et al. 2001) and ACS (Matsuo et al. 1998; Yoshida et al. 2013). Cardiac BMIPP SPECT can show more accumulation defects in damaged myocardium than cardiac perfusion SPECT due to the slow recovery of myocardial fatty-acid metabolism impairment in patients with myocardial damage, including TC and ACS (Miyachi et al. 2013; Hambye et al. 2000; Ito et al. 2005). However, since SPECT-only imaging does not show coronary artery distribution, the diagnostic performance of this modality is not sufficient. In addition, simultaneous use of ^99m^Tc perfusion tracers, such as ^99m^Tc-methoxy-isobutyl-isonitrile (MIBI), and BMIPP under conventional NaI gamma cameras has still been limited due to their mutual crosstalk rates.

Hybrid cardiac SPECT/computed tomography (CT) imaging simultaneously shows myocardial damage distribution and coronary artery distribution as well as their relationship. This imaging can easily be performed using stand-alone systems with dedicated fusion software (Gaemperli et al. 2007, 2007, 2008; Rispler et al. 2007). Several reports have shown that hybrid cardiac SPECT/CT imaging has a higher diagnostic performance than SPECT-only imaging for CAD (Gaemperli et al. 2007, 2008; Rispler et al. 2007). Similarly, hybrid cardiac SPECT/CT may have higher diagnostic performance compared with SPECT-only imaging for the differential diagnosis of TC or ACS (Miyachi et al. 2013).

The aims of this study were to reveal the feasibility of dual-isotope MIBI and BMIPP imaging using conventional NaI gamma cameras, and to evaluate the diagnostic performance of hybrid cardiac perfusion and fatty-acid metabolism SPECT/CT imaging compared with SPECT-only imaging for patients with TC.

## Methods

### Phantom study for dual-isotope MIBI and BMIPP SPECT imaging

Simulating MIBI and BMIPP dual-isotope SPECT imaging, the mutual crosstalk rates of ^99m^Tc and ^123^I were measured using an RH-2 type cardiac phantom (Kyoto Kagaku, Kyoto, Japan) to determine the most suitable energy windows for the dual-isotope acquisition. The cardiac phantom was filled with ^99m^Tc: 6.66 MBq/175 ml (38.06 kBq/ml) for one acquisition, and ^123^I: 5.99 MBq/175 ml (34.23 kBq/ml) for the other. The doses of radioactive tracers were determined based on reports about biodistribution of MIBI and BMIPP (Kubo et al. 1992; Torizuka et al. 1991).

SPECT data acquisitions of the phantoms were performed 60 min after the construction of the phantom model using a dual-headed gamma camera: Infinia (GE Healthcare Japan, Tokyo, Japan). The energy windows at 140 keV were set at the ranges −5% to +5%, −6% to +4%, −7% to +3%, −8% to +2%, and −9% to +1%, and those at 159 keV were set at the ranges −5% to +5%, −4% to +6%, −3% to +7%, −2% to +8%, and −1% to +9%. Within these energy windows, gamma-ray counts of ^99m^Tc and ^123^I were then measured, respectively. Within the 140 keV windows, crosstalk rates of ^123^I were calculated dividing the radioactive count of ^123^I by that of ^99m^Tc; within the 159 keV windows, the crosstalk rates of ^99m^Tc were calculated dividing the radioactive count of ^99m^Tc by that of ^123^I. For all tests, 30 projection images were obtained in 6-degree increments over an orbit of 180 degrees at a rate of 45 s per projection. The image matrix size was 64, and an extended low energy, general-purpose collimator was used.

### Clinical study

#### Patient population

**Fig. 1:**
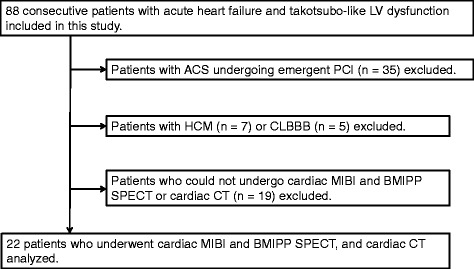
Flow chart of patient inclusion and exclusion in this study

A total of 88 consecutive patients with acute heart failure and apical-ballooning type LV dysfunction, admitted to the coronary care unit between January 2010 and June 2016, were included in this study. Patients with ACS who underwent emergent percutaneous coronary intervention (PCI) (*n* = 35) were excluded. Furthermore, patients with hypertrophic cardiomyopathy (*n* = 7) and those with complete left bundle branch block (*n* = 5) were also excluded. Out of the remaining 41 patients, 22 patients who underwent cardiac dual-isotope MIBI and BMIPP SPECT and cardiac CT (CCT) were analyzed [8 men and 14 women, 74 (66–84) years] (Fig. 1). Clinical characteristics of the final study cohort are shown in Table 1. The study protocol was received and approved by the institutional review board, and written informed consent was obtained from all study participants.

**Table 1:** Patient characteristics

Number of patients	22
Age (years)	74 (66–84)
Female	14 (64%)
Coronary risk factor	
Smoking habit	9 (41%)
Hypertension	18 (82%)
Dyslipidemia	10 (45%)
Diabetes mellitus	5 (23%)
Family history	3 (14%)
Blood exam	
CK (IU/l)	140 (98–373)
CK-MB (ng/ml)	10 (6–21)
Troponin T (ng/dl)	0.051 (0.020–0.599)
BNP (pg/ml)	185 (61–501)
Onset to SPECT (days)	11 (8–15)
Onset to CCT (days)	9 (6–14)

*CK* Creatine kinase, *BNP* Brain natriuretic peptide

#### Cardiac dual-isotope MIBI and BMIPP SPECT imaging

All analyzed participants underwent a cardiac dual-isotope MIBI and BMIPP SPECT test. SPECT data were acquired 60 min after the simultaneous intravenous administration of MIBI 600 MBq and BMIPP 148 MBq using the same method as in the phantom study, with the addition of ECG-gated acquisition. MIBI and BMIPP data were acquired using the most suitable energy window for each radioactive tracer derived from the phantom study. Collected data were reformatted into non-ECG-gated short axial, horizontal long axial, and vertical long axial SPECT images via reconstruction with ordered-subsets expectation maximization, without attenuation correction, using an iteration of 4 and subset of 10.

#### CCT imaging

All CT scans were performed on a 64-section CT scanner: Light Speed VCT (GE Healthcare Japan, Tokyo, Japan) with helical scanning and retrospective ECG-gated reconstruction. An optimal dose of metoprolol was administered orally 90 min before CT scans to achieve target heart rate (< 65 beats/min). CT data were obtained using bolus tracking method after an intravenous injection of 0.8 ml/kg of iodine contrast material Iopamiron 370 (Bayer Healthcare Pharmaceuticals, Tokyo, Japan) and reconstructed into 0.625-mm slice transaxial images, multiplanar reformatted images, and maximum-intensity projection images.

#### Hybrid cardiac SPECT/CT imaging

For all analyzed patients, the fused images of cardiac dual-isotope MIBI and BMIPP SPECT with CCT were created using a dedicated software, CardIQ Fusion (GE Healthcare Japan, Tokyo, Japan), installed on a diagnostic image viewer. This software allows the projection of cardiac SPECT images onto the LV epicardial surface of CCT images and the creation of 3D volume-rendered hybrid cardiac SPECT/CT images.

#### Data acquisition

Image interpretations of cardiac SPECT, CT, and hybrid SPECT/CT images were performed under the consensus of two experienced nuclear medicine specialists with no preexisting knowledge of the other modality findings.

#### Cardiac SPECT

**Fig. 2:**
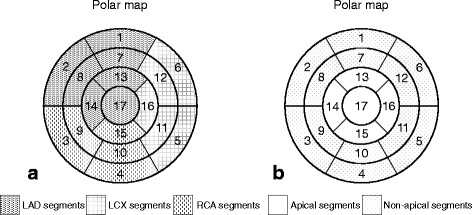
Left ventricular myocardium polar maps based on 17-segment model. Most standard coronary artery distribution segments (**a**), and apical and non-apical segments (**b**)

Accumulation defects were visually quantified using a 17-segment model of the LV with a 5-point grading system (0, normal uptake; 1, slightly-reduced uptake; 2, moderately-reduced uptake; 3, severely-reduced uptake; and 4, absent uptake) (Fig. 2a) (Cerqueira et al. 2002). Summed MIBI and BMIPP defect scores (SMDS and SBDS) were calculated by summating the scores in all segments. SMDS and SBDS reflect the severity of myocardial perfusion and fatty-acid metabolism impairment, respectively. The summed mismatch score (SMS) was then calculated by deducting SMDS from SBDS. SMS reflects the severity of acute myocardial damage, commonly caused by myocardial ischemia. In order to prevent underestimation of the accumulation in the LV apical wall, the accumulation and thickness were compared between end-diastolic and end-systolic images to distinguish true myocardial damage from apical thinness. Furthermore, apical MIBI and BMIPP defect scores (A-MDS and A-BDS) and non-apical MIBI and BMIPP defect scores (NA-MDS and NA-BDS) were calculated by summating the defect scores in segments 13–17 and segments 1–12, respectively (Fig. 2b). The apical mismatch score (A-MS) and non-apical mismatch score (NA-MS) were also calculated using the same procedure as with SMS.

Diagnoses by SPECT-only imaging were determined based on the most standard coronary artery territories. The diagnoses were categorized as TC: SPECT accumulation defects around apical LV wall deviated from the most standard coronary artery territories (Fig. 2a), equivocal: unclear relationship of accumulation defects and coronary artery territories, or non-TC: accumulation defects coincided with coronary artery territories. In the ECG-gated MIBI SPECT images, LV ejection fraction, LV end-diastolic volume, LV end-systolic volume, and the percentages of wall thickening in the apical and non-apical wall (A-%WT and NA-%WT) were calculated using Quantitative Gated SPECT (Cedars Sinai Medical Center, Los Angeles, USA).

#### CCT

The presence or absence of coronary artery stenoses was assessed according to the Society of Cardiovascular Computed Tomography coronary segmentation diagram (Leipsic et al. 2014). A diameter stenosis ≥70% was considered a significant coronary lesion. The findings of CCT were compared with those of ICA to confirm the CCT findings.

#### Hybrid cardiac SPECT/CT

Diagnoses by hybrid cardiac SPECT/CT imaging were determined based on the accordance of the locations of myocardial damage and coronary artery territories. The diagnoses were categorized as TC: SPECT accumulation defects around apical LV wall deviated from the actual coronary artery territories, equivocal: unclear relationship of accumulation defects and the coronary artery territories, or non-TC: accumulation defects coincided with the coronary artery territories.

### Diagnostic confirmation of TC

Definitive diagnoses of TC, as opposed to ACS, were confirmed under the consensus of two experienced cardiologists according to the proposed diagnostic criteria for TC, including (1) sudden occurrence of heart failure, (2) transient systolic dysfunction of the LV apical segments extending beyond a single coronary territory, (3) absence of significant (≥ 70%) obstructive coronary artery disease excluding preexisting conditions, and (4) complete normalization of the LV dysfunction confirmed by echocardiography three or more weeks after onset (Kawai et al. 2001). Diagnostic accuracies of SPECT-only analysis and SPECT/CT fused analysis were evaluated based on the definitive diagnoses of TC.

### Statistical analysis

As all continuous variables were not distributed normally, the data were expressed as medians with 25th and 75th percentiles. Categorical variables were presented as counts (%).

In a comparison of clinical profiles between TC and ACS groups, non-normally distributed continuous variables were compared using a Mann-Whitney U-test, and categorical variables were compared using a Mann-Whitney U-test or a Fisher’s exact probability test. The difference in image interpretation (TC, equivocal, or non-TC) between SPECT-only and SPECT/CT fused analyses was evaluated using a χ^2^ test with William’s correction.

A *p*-value <0.05 was considered statistically significant. All statistical analyses were performed using StatMate IV software version 4.01 (Advanced Technology for Medicine and Science, Tokyo, Japan).

## Results

### Phantom study

**Fig. 3:**
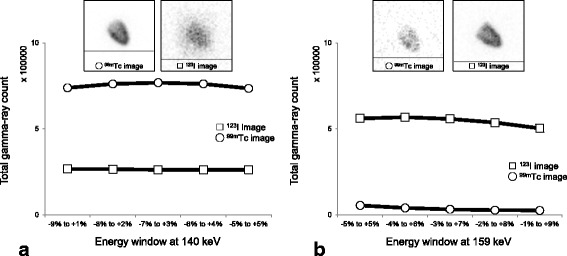
Total gamma-ray counts of ^99m^Tc (white circles) and ^123^I (white squares) in each energy window. Within 140 keV windows (**a**). Within 159 keV windows (**b**)

Acquired gamma-ray counts of ^99m^Tc and ^123^I at each energy window are shown in Fig. 3. Maximum values for the counts of ^99m^Tc in the 140 keV energy window and ^123^I in the 159 keV energy window were at ranges −7% to +3% and −4% to +6%, respectively. In the 140 keV energy windows, the crosstalk rates of ^123^I within ranges of −5% to +5%, −6% to +4%, −7% to +3%, −8% to +2%, and −9% to +1% were 26.3%, 25.6%, 25.4%, 25.9%, and 26.6%, respectively. In the 159 keV energy windows, the crosstalk rates of ^99m^Tc within ranges of −5% to +5%, −4% to +6%, −3% to +7%, −2% to +8%, and −1% to +9% were 8.9%, 6.6%, 5.3%, 4.8%, and 4.7%, respectively. Based on these results, the most suitable window ranges for ^99m^Tc at 140 keV and for ^123^I at 159 keV were confirmed to be −7% to +3% and −4% to +6%, respectively.

### Clinical study

#### Patient population

Twenty-two patients with apical-ballooning type acute heart failure who underwent cardiac dual-isotope MIBI and BMIPP SPECT test and CCT were included in the analyses (Table 1). As determined by the diagnostic criteria for TC, both the TC and ACS groups consisted of 11 participants. The clinical characteristics of the two groups are presented in Table 2.

**Table 2:** Patient characteristics in TC and ACS groups

	TC (*n* = 11)	ACS (n = 11)	*P* value
Age (years)	75 (69–86)	72 (63–81)	0.216
Female	8 (73%)	6 (55%)	0.658
Coronary risk factor			
Smoking habit	1 (9%)	8 (73%)	0.009
Hypertension	10 (90%)	8 (73%)	0.580
Dyslipidemia	2 (18%)	8 (73%)	0.032
Diabetes mellitus	2 (18%)	3 (27%)	1.000
Family history	0 (0%)	3 (27%)	0.214
Blood exam			
CK (IU/l)	122 (94–155)	298 (114–540)	0.177
CK-MB (ng/ml)	9 (6–17)	12 (6–23)	0.533
Troponin T (ng/dl)	0.038 (0.016–0.280)	0.052 (0.041–0.834)	0.503
BNP (pg/ml)	176 (119–695)	191 (44–327)	0.514
Onset to SPECT (days)	10 (7–14)	13 (9–18)	0.289
Onset to CCT (days)	8 (6–10)	9 (7–18)	0.503

*CK* Creatine kinase, *BNP* Brain natriuretic peptide

#### Cardiac SPECT findings

SMDS was 5 (3–7) for patients with TC and 8 (4–16) for patients with ACS (*p* = 0.216). SBDS was 10 (8–12) in the TC group and 18 (9–24) in the ACS group (*p* = 0.354) (Table 3). AMDS and ABDS did not differ between TC and ACS patients for (*p* = 0.567 and *p* = 0.386, respectively), while NA-MDS and NA-BDS were significantly higher for patients with ACS (*p* = 0.022 and *p* = 0.022, respectively) (Table 3). Both A-%WT and NA-%WT did not significantly differ between TC and ACS patients (*p* = 0.546 and *p* = 0.302, respectively) (Table 3).

**Table 3:** Cardiac SPECT and CT findings

	TC (*n* = 11)	ACS (*n* = 11)	*P* value
Cardiac SPECT			
SMDS	5 (3–7)	8 (4–16)	0.216
A-MDS	3 (2–6)	3 (1–7)	0.567
NA-MDS	0 (0–2)	5 (3–7)	0.022
SBDS	10 (8–12)	18 (9–24)	0.354
A-BDS	8 (5–11)	7 (2–10)	0.386
NA-BDS	0 (0–5)	9 (6–13)	0.022
SMS	5 (3–7)	6 (3–8)	0.531
A-MS	3 (2–6)	1 (1–3)	0.136
NA-MS	0 (0–2)	3 (2–6)	0.080
ECG-gated cardiac SPECT			
LVEF (%)	69 (54–74)	62 (43–71)	0.503
LVEDV (ml)	69 (62–83)	93 (58–119)	0.497
LVESV (ml)	23 (16–39)	35 (20–65)	0.460
A-%WT	48 (32–56)	48 (27–57)	0.546
NA-%WT	39 (28–45)	28 (21–44)	0.302
Cardiac CT			
CAD (none/1VD/2VD/3VD)	7/2/1/1	5/3/1/2	0.900
LAD stenosis	3 (27%)	3 (27%)	1.000
Dx stenosis	1 (9%)	3 (27%)	0.586
LCX stenosis	1 (9%)	5 (45%)	0.149
RCA stenosis	2 (18%)	3 (36%)	1.000

*SMDS* Summed MIBI defect score, *A-MDS* Apical MIBI defect score, *NA-MDS* Non-apical MIBI defect score, *SBDS* Summed BMIPP defect score, *A-BDS* Apical BMIPP defect score, *NA-BDS* Non-apical BMIPP defect score, *SMS* Summed mismatch score, *A-MS* Apical mismatch score, *NA-MS* Non-apical mismatch score, *LVEF* Left ventricular ejection fraction, *LVEDV* Left ventricular end-diastolic volume, *LVESV* Left ventricular end-systolic volume, *A-%WT* Apical % wall thickening, *NA-%WT* Non-apical % wall thickening, *CAD* Coronary artery disease, *VD* Vessel disease

#### CCT findings

A total of 334 coronary artery segments in 22 patients were assessed by CCT and the results are shown in Table 3. ICA was performed in all patients. In 19 out of 22 patients, the diagnosis of significant coronary lesion obtained from CCT was consistent with that obtained from ICA, indicating the excellent diagnostic performance of CCT for patients with TC or ACS. Compared with ICA, the sensitivity, specificity, positive predictive value, and negative predictive value of CCT were 92%, 99%, 88%, and 93%, respectively.

#### Diagnoses with SPECT-only and SPECT/CT fused analyses

**Fig. 4:**
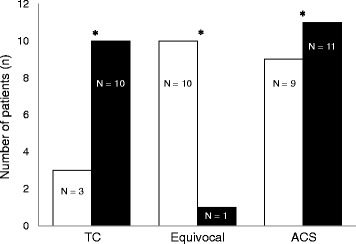
Alteration of image interpretation (TC or non-TC) between SPECT-only (white columns) and SPECT/CT fused (black columns) analyses

All patients underwent hybrid cardiac SPECT/CT imaging. Out of the 10 patients with equivocal results on SPECT-only analysis, 7 were diagnosed with TC and 2 were diagnosed with ACS based on SPECT/CT fused analysis. Consequently, the number of equivocal results was significantly decreased by SPECT/CT fused analysis (*p* = 0.040) (Fig. 4). Concerning the diagnosis of TC, SPECT/CT fused analysis showed a superior diagnostic performance (sensitivity, 90%; specificity, 100%; and accuracy, 95%) compared with SPECT-only analysis (sensitivity, 30%; specificity, 81%; and accuracy, 57%) (Table 4).

**Table 4:** Diagnostic accuracies of SPECT-only imaging and hybrid SPECT/CT imaging

	Sensitivity (%)	Specificity (%)	Accuracy (%)
SPECT-only imaging	30	81	57
Hybrid SPECT/CT imaging	90	100	95

#### Case presentations

**Fig. 5:**
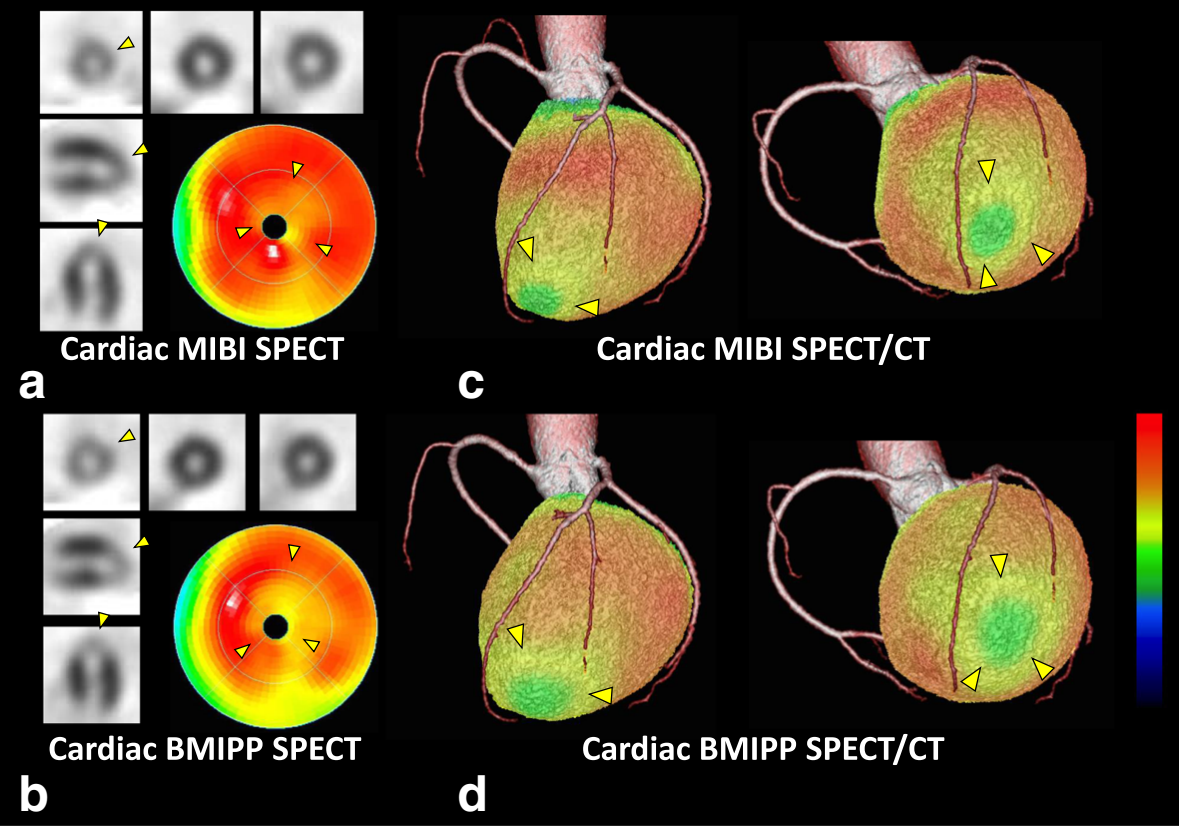
68-year-old woman with TC. Cardiac MIBI and BMIPP SPECT images show accumulation defects in the distal anterolateral to apical wall (**a**, **b**). Due to the unclear alignment of accumulation defects with coronary artery territories, SPECT-only imaging could not differentiate between TC and ACS. SPECT/CT fused images revealed that the accumulation defect extended through distal LAD and first diagonal branch territories, confirming TC (**c**, **d**)

Figure 5 shows a representative case of a patient with TC. In this 68-year-old woman with chest pain, negative T in V3–6 and ST-depression in V5 and V6 were observed on ECG upon admission. A blood test showed slightly high troponin T (0.038 ng/ml) and CCT showed no coronary artery stenoses. Cardiac MIBI and BMIPP SPECT were performed 8 days after the onset. MIBI images showed slightly reduced accumulation in the distal anterolateral to apical wall in the LV myocardium, while BMIPP images showed moderately reduced accumulation in the same region. Since the myocardial damage only appears to be slightly congruent with typical diagonal branch territory, SPECT-only imaging could not differentiate between TC and ACS. In the SPECT/CT fused images, the accumulation defect extended through distal LAD and first diagonal branch territories and this patient was therefore diagnosed with TC.

**Fig. 6:**
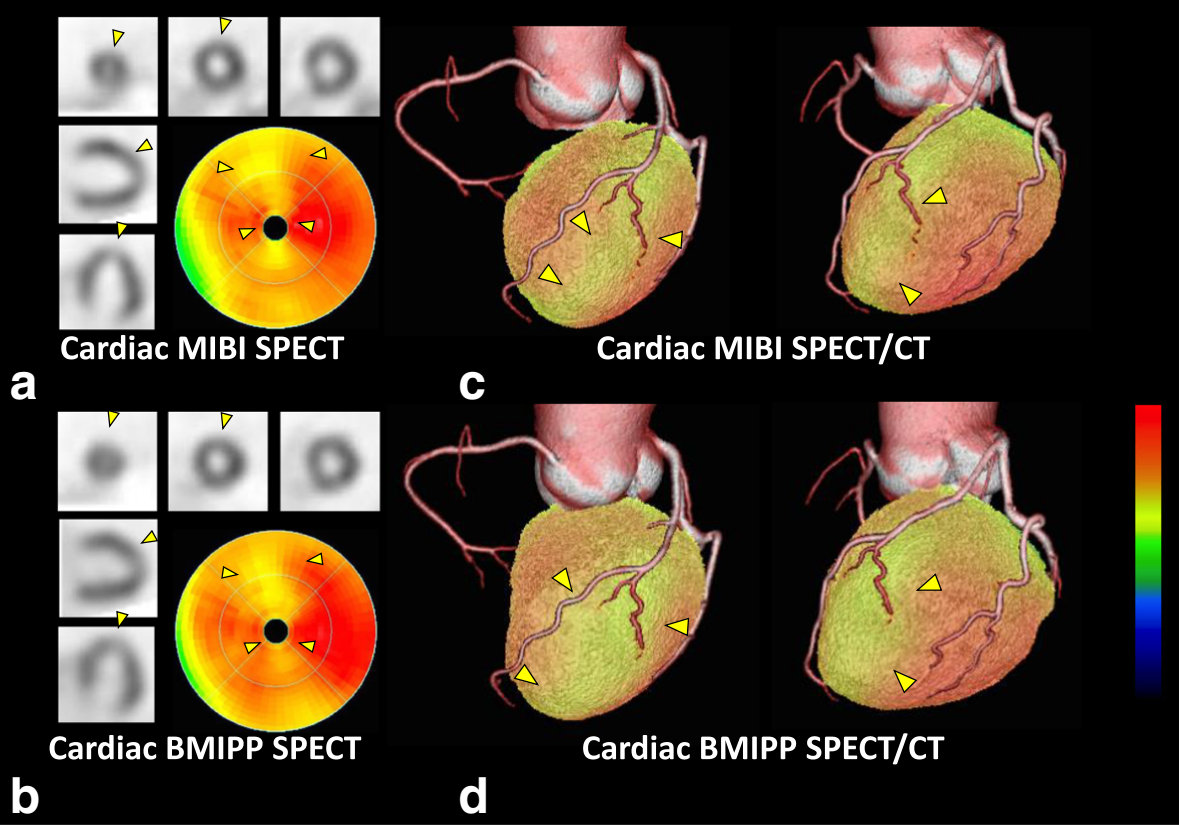
60-year-old woman with ACS. Cardiac MIBI and BMIPP SPECT images show accumulation defects in the mid-distal anteroseptal to apical wall and this patient may have ACS in the LAD territory (**a**, **b**). SPECT/CT fused images revealed that the accumulation defect is congruent with first diagonal branch territory, confirming ACS due to coronary vasospasm (**c**, **d**)

Figure 6 shows a typical case of a patient with ACS. In this 60-year-old woman with chest pain, ST-depression in V4–6 was observed on ECG. A blood test showed slightly high troponin T (0.052 ng/ml) and CCT showed no coronary artery stenoses. Cardiac MIBI and BMIPP SPECT were performed 7 days after the onset. MIBI images showed slightly reduced accumulation in the anterior to apical wall in the LV myocardium, while BMIPP images showed slightly to moderately reduced accumulation in the same region. Since the myocardial damage appeared to be congruent with typical LAD territory, this patient was thought to have ACS in LAD territory. In the SPECT/CT fused images, the accumulation defect was congruent with first diagonal branch territory, and this patient was therefore diagnosed with ACS due to first diagonal branch vasospasm.

## Discussion

In this study, the feasibility of dual-isotope MIBI and BMIPP imaging using conventional NaI gamma cameras and the diagnostic performance of hybrid cardiac SPECT/CT imaging for patients with TC was examined. The results showed that the crosstalk rates of ^123^I at 140 keV and of ^99m^Tc at 159 keV were 25.4% and 6.6%, respectively, and SPECT/CT fused analysis had improved diagnostic performance compared with SPECT-only analysis.

### Cardiac SPECT for diagnosing TC

ICA is the most common modality for diagnosing TC, eliminating obstructive coronary artery lesions. In contrast, cardiac perfusion and fatty-acid metabolism SPECT are applied for diagnosing TC, detecting the myocardial damage instead. However, cardiac dual-isotope MIBI and BMIPP imaging using conventional NaI gamma cameras has been limited for use even though several investigators reported positively about the imaging procedures (Yoshida et al. 2013; Hirata et al. 2009). Recently, the dual-isotope SPECT imaging technique using semiconductor gamma cameras was introduced. The crosstalk rate of ^123^I at 140 keV and that of ^99m^Tc at 159 keV using a semiconductor gamma camera are reported to be >20% and <1%, respectively (Takahashi et al. 2015). Varying slightly, in this study, using an NaI gamma camera and suitable energy windows, the crosstalk rate of ^123^I at 140 keV and that of ^99m^Tc at 159 keV were 25.4% and 6.6%, respectively. While semiconductor detectors have superior energy resolution and sensitivity compared with NaI gamma cameras, this study shows that dual-isotope SPECT imaging using an NaI gamma camera is feasible without significant influence on the diagnosis. Using this method, diagnosing TC using cardiac dual-isotope SPECT will be more accessible, even in facilities that do not have a semiconductor detector.

For the clinical study, the patients with TC underwent both cardiac MIBI and BMIPP SPECT tests 10 (7–14) days from the onset of TC, mostly within the subacute phase. Ito et al. reported that SBDS was significantly higher than summed perfusion score (3.2 ± 3.0 vs. 12.6 ± 3.7) (Ito et al. 2005). In this study, SBDS was significantly higher than SMDS (5 (3–7) vs. 10 (8–12), respectively, *p* = 0.003) similarly to Ito et al. Furthermore, myocardial regional accumulation of MIBI and BMIPP was observed in this study. While A-MDS and A-BDS did not differ between TC and ACS, NA-MDS and NA-BDS were higher in ACS than TC. However, Matsuo et al. reported that both A-BDS and NA-BDS were significantly higher in ACS than TC. The discrepancy of the results between Matsuo et al. and this study is due to the difference in the populations of the ACS groups, which of this study had myocardial ischemia exclusively in the distal LAD territory, resulting in a comparatively mild myocardial ischemia (Matsuo et al. 2014).

### CCT for diagnosing TC

ICA is commonly performed for diagnosing TC (Ito et al. 2003). However, this invasive procedure may not necessarily be required for patients with a low pretest probability of ACS. In this study, the findings from CCT and ICA were strongly correlated and the diagnostic accuracy of CCT was within the range of previously published data (Gaemperli et al. 2007). As an alternative, CCT can reveal obstructive coronary artery lesions even in patients with suspected TC.

In this study, 4 out of 11 patients in the TC group (36%) had significant coronary artery stenoses and no ischemic myocardial damage in the corresponding coronary artery territories. Patients with TC and incidental significant coronary artery stenoses have the risk of being misdiagnosed with ACS. Also, 5 out of 11 patients in the ACS group (45%) did not have significant coronary artery stenoses and were diagnosed with ACS caused by coronary vasospasm via SPECT/CT imaging. In the general clinical setting, patients with ACS often have no significant coronary artery stenoses (Libby and Theroux 2005), and identification of the ischemic myocardial damage with cardiac SPECT is preferable for such patients (Hambye et al. 2000).

### Clinical application of hybrid cardiac SPECT/CT imaging for TC

Hybrid cardiac SPECT/CT imaging can easily be conducted with a stand-alone system and dedicated fusion software, directly correlating the distribution of LV myocardial damage with the distribution of coronary arteries. Several studies have revealed that the use of hybrid cardiac SPECT/CT imaging increased the conclusive diagnostic rate compared with side-by-side interpretations of SPECT and CCT images in patients with CAD (Gaemperli et al. 2007, 2008; Rispler et al. 2007). In this study, hybrid cardiac SPECT/CT imaging was also used for diagnosing TC. While the indeterminate diagnostic rate for TC was remarkably high in the SPECT-only analysis (45%), the indeterminate diagnostic rate significantly decreased (5%) in the SPECT/CT fused analysis. Hybrid cardiac SPECT/CT imaging shows the relationship between the distributions of myocardial damage and coronary arteries and thus the cause of myocardial damage, TC or ACS, can easily be resolved. The detailed relationship of the distributions indicated by this hybrid imaging also permitted the differentiation of TC with ACS from solely ACS. Therefore, hybrid cardiac SPECT/CT imaging may help prevent the misdiagnosis of these patients.

### Study limitations

The main limitation of this study was the small patient sample size of 22 in total, thus limiting statistical reliability. Although NA-MDS and NA-BDS differed between the TC and ACS groups, several parameters, such as SMDS, NA-MDS, SBDS, and NA-BDS, have the potential to be higher in the ACS group as shown in previous studies. A further limitation of the study was that the cardiac SPECT images were reconstructed without attenuation correction. In the hybrid cardiac SPECT/CT images, attenuation artifacts, especially in the inferoposterior wall, are conspicuous and have the potential to be misdiagnosed as true myocardial damage. Nonetheless, the SPECT tests in this study were all performed by ECG-gated acquisition and the artifacts could be discriminated. The imaging periods in this study were mostly within the subacute phase and the interval between onset and SPECT was slightly longer in the ACS group than the TC group. This may have allowed for a reduction of defect scores in the ACS group. Despite these limitations, the results of this study show a clear pattern on the diagnostic performance of hybrid cardiac SPECT/CT which is likely applicable in a wide clinical setting. Furthermore, this hybrid cardiac SPECT/CT imaging could also be applied to diagnose other causes of congestive heart failure by eliminating ischemic heart failure.

## Conclusions

In this study, the feasibility of dual-isotope MIBI and BMIPP imaging using conventional NaI gamma cameras was revealed, resulting from the crosstalk rates of ^123^I at 140 keV and ^99m^Tc at 159 keV of 25.4% and 6.6%, respectively. Furthermore, hybrid cardiac SPECT/CT imaging using MIBI and BMIPP allows comprehensive assessment of coronary artery distribution and myocardial damage distribution and may have superior diagnostic performance compared with SPECT-only imaging for patients with TC.

## Abbreviations

A-BDS: Apical BMIPP defect score
ACS: Acute coronary syndrome
A-MDS: Apical MIBI defect score
A-MS: Apical mismatch score
Apical: A-%WT and non-apical % wall thickening = NA-%WT
BMIPP: ^123^I–beta-methyl-P-iodophenyl-pentadecanoic acid
CAD: Coronary artery disease
CCT: Cardiac CT
CT: Computed tomography
ECG: Electrocardiograms
ICA: Invasive coronary angiography
LAD: Left anterior descending artery
LV: Left ventricular
MIBI: ^99m^Tc-methoxy-isobutyl-isonitrile
NA-BDS: Non-apical BMIPP defect score
NA-MDS: Non-apical MIBI defect score
NA-MS: Non-apical mismatch score
PCI: Percutaneous coronary intervention
SBDS: Summed BMIPP defect score
SMDS: Summed MIBI defect score
SMS: Summed mismatch score
SPECT: Single-photon emission computed tomography
TC: Takotsubo cardiomyopathy

## References

[CR1] Barletta G, Del Pace S, Boddi M, Del Bene R, Salvadori C, Bellandi B (2009). Abnormal coronary reserve and left ventricular wall motion during cold pressor test in patients with previous left ventricular ballooning syndrome. Eur Heart J.

[CR2] Cerqueira MD, Weissman NJ, Dilsizian V, Jacobs AK, Kaul S, Laskey WK (2002). Standardized myocardial segmentation and nomenclature for tomographic imaging of the heart: a statement for healthcare professionals from the cardiac imaging Committee of the Council on clinical cardiology of the American Heart Association. Circulation.

[CR3] Gaemperli O, Schepis T, Kalff V, Namdar M, Valenta I, Stefani L (2007). Validation of a new cardiac image fusion software for three-dimensional integration of myocardial perfusion SPECT and stand-alone 64-slice CT angiography. Eur J Nucl Med Mol Imaging.

[CR4] Gaemperli O, Schepis T, Valenta I, Husmann L, Scheffel H, Duerst V (2007). Cardiac image fusion from stand-alone SPECT and CT: clinical experience. J Nucl Med.

[CR5] Gaemperli O, Schepis T, Valenta I, Koepfli P, Husmann L, Scheffel H (2008). Functionally relevant coronary artery disease: comparison of 64-section CT angiography with myocardial perfusion SPECT. Radiology.

[CR6] Hachamovitch R, Hayes SW, Friedman JD, Cohen I, Berman DS (2003). Comparison of the short-term survival benefit associated with revascularization compared with medical therapy in patients with no prior coronary artery disease undergoing stress myocardial perfusion single photon emission computed tomography. Circulation.

[CR7] Hambye AS, Vervaet A, Dobbeleir A, Dendale P, Franken P (2000). Prediction of functional outcome by quantification of sestamibi and BMIPP after acute myocardial infarction. Eur J Nucl Med.

[CR8] Hirata M, Monzen H, Suzuki T, Ogasawara M, Nakanishi A, Sumi N (2009). Evaluation of a new protocol for two-isotope ^123^I-BMIPP/^99m^Tc-TF single photon emission computed tomography (SPECT) to detect myocardial damage within one hour. Igaku Butsuri.

[CR9] Ito K, Sugihara H, Katoh S, Azuma A, Nakagawa M (2003). Assessment of Takotsubo (ampulla) cardiomyopathy using ^99m^Tc-tetrofosmin myocardial SPECT--comparison with acute coronary syndrome. Ann Nucl Med.

[CR10] Ito K, Sugihara H, Kinoshita N, Azuma A, Matsubara H (2005). Assessment of Takotsubo cardiomyopathy (transient left ventricular apical ballooning) using 99mTc-tetrofosmin, ^123^I-BMIPP, ^123^I-MIBG and ^99m^Tc-PYP myocardial SPECT. Ann Nucl Med.

[CR11] Kawai S, Kitabatake A, Tomoike H (2007). Takotsubo cardiomyopathy study group. Guidelines for diagnosis of takotsubo (ampulla) cardiomyopathy. Circ J.

[CR12] Kawai S, Suzuki H, Yamaguchi H, Tanaka K, Sawada H, Aizawa T (2000). Ampulla cardiomyopathy (‘Takotusbo’ cardiomyopathy) --reversible left ventricular dysfunction with ST segment elevation. Jpn Circ J.

[CR13] Kawai Y, Tsukamoto E, Nozaki Y, Morita K, Sakurai M, Tamaki N (2001). Significance of reduced uptake of iodinated fatty acid analogue for the evaluation of patients with acute chest pain. J Am Coll Cardiol.

[CR14] Kubo A, Nakamura K, Hashimoto J, Sammiya T, Iwanaga S, Hashimoto S (1992). Phase I clinical trial of a new myocardial imaging agent, ^99m^Tc-PPN1011. Kaku Igaku.

[CR15] Kurisu S, Inoue I, Kawagoe T, Ishihara M, Shimatani Y, Nishioka K (2003). Myocardial perfusion and fatty acid metabolism in patients with tako-tsubo-like left ventricular dysfunction. J Am Coll Cardiol.

[CR16] Kurisu S, Kihara Y (2012). Tako-tsubo cardiomyopathy: clinical presentation and underlying mechanism. J Cardiol.

[CR17] Leipsic J, Abbara S, Achenbach S, Cury R, Earls JP, Mancini GJ (2014). SCCT guidelines for the interpretation and reporting of coronary CT angiography: a report of the Society of Cardiovascular Computed Tomography Guidelines Committee. J Cardiovasc Comput Tomogr.

[CR18] Libby P, Theroux P (2005). Pathophysiology of coronary artery disease. Circulation.

[CR19] Matsuo S, Nakajima K, Kinuya S, Yamagishi M (2014). Diagnostic utility of ^123^I-BMIPP imaging in patients with Takotsubo cardiomyopathy. J Cardiol.

[CR20] Matsuo S, Nakamura Y, Takahashi M, Mitsunami K, Kinoshita M (1998). Myocardial metabolic abnormalities in hypertrophic cardiomyopathy assessed by iodine-123-labeled beta-methyl-branched fatty acid myocardial scintigraphy and its relation to exercise-induced ischemia. Jpn Circ J.

[CR21] Miyachi H, Kumita S, Tanaka K (2013). PET/CT and SPECT/CT cardiac fusion imaging in a patient with takotsubo cardiomyopathy. Eur Heart J.

[CR22] Prasad A, Lerman A, Rihal CS (2008). Apical ballooning syndrome (Tako-Tsubo or stress cardiomyopathy): a mimic of acute myocardial infarction. Am Heart J.

[CR23] Rispler S, Keidar Z, Ghersin E, Roguin A, Soil A, Dragu R (2007). Integrated single-photon emission computed tomography and computed tomography coronary angiography for the assessment of hemodynamically significant coronary artery lesions. J Am Coll Cardiol.

[CR24] Takahashi Y, Miyagawa M, Nishiyama Y, Kawaguchi N, Ishimura H, Mochizuki T (2015). Dual radioisotopes simultaneous SPECT of ^99m^Tc-tetrofosmin and ^123^I-BMIPP using a semiconductor detector. Asia Ocean J Nucl Med Biol.

[CR25] Tamaki N, Kawamoto M, Yonekura Y, Fujibayashi Y, Takahashi N, Konishi J (1992). Regional metabolic abnormality in relation to perfusion and wall motion in patients with myocardial infarction: assessment with emission tomography using an iodinated branched fatty acid analog. J Nucl Med.

[CR26] Torizuka K, Yonekura Y, Nishimura T, Tamaki N, Uehara T, Ikekubo K (1991). A phase 1 study of betamethyl-p-(^123^I)-iodophenyl-pentadecanoic acid (^123^I-BMIPP). Kaku Igaku.

[CR27] Tsuchihashi K, Ueshima K, Uchida T, Oh-mura N, Kimura K, Owa M (2001). Transient left ventricular apical ballooning without coronary artery stenosis: a novel heart syndrome mimicking acute myocardial infarction. Angina pectoris-myocardial infarction investigations in Japan. J Am Coll Cardiol.

[CR28] Yoshida A, Takano H, Asai K, Yasutake M, Amano Y, Kumita S (2013). Comparison of perfusion-metabolism mismatch in ^99m^Tc-MIBI and ^123^I-BMIPP scintigraphy with cardiac magnetic resonance in patients with dilated cardiomyopathy. J Card Fail.

